# Gun-shot Wound—Ball Opening the Gravid Uterus—Death in Twenty Hours

**Published:** 1848-09

**Authors:** Paul F. Eve

**Affiliations:** Professor of Surgery in the Medical College of Georgia


*Gun-shot wound—ball opening the gravid uturus—death in twenty hours.* By Paul F. Eve, M. D., Professor of Surgery in the Medical College of Georgia.—At 1 o'clock, A. M., March 20th, 1838, I was called up to see Charlotte, a negro girl aged 19 years, said to have been accidentally shot. On arriving, I found Dr. R. in attendance, and learned that our patient had just received a pistol ball at the distance of about six paces. She was engaged with a large dancing party when interrupted by the accident. She was standing near the fireplace at the time; the ball entered just above the anterior superior spinous process of the ileum, and penetrated transversely the hypogastric region. The probe and finger could follow in its tract, without, however, ascertaining where it had lodged or whether it had opened the abdomen. There was no external hemorrhage nor intestinal protrusion. Besides the shock to the general system, there were no special symptoms beyond the ordinary appearance of a gun-shot wound. Upon inquiry, we learned that Charlotte was about the fourth month of utero-gestation.

At 4 o'clock a loop of the bowels protruded through the external wound, which was immediately returned by taxis, and then retained by compress and bandage. Our patient now exhibited symptoms of prostration, which continued to increase, notwithstanding the means employed to counteract them, and she died at 8 P. M.—twenty hours after she was shot.

Early the next morning, 21st March, in presence of the Medical Class, the abdomen was laid open by a crucial incision. About half a gallon of clotted blood was removed from the pelvis, and a *small fœtus with its secundines.* The ball was found to have penetrated the abdominal parietes, passing through two or three convolutions of the small intestines, without producing any escape of their contents; it then made quite a large, ragged opening into the uterus, at its anterior superior portion of the fundus, and striking the left iliac fossa, dropped into the cavity of the pelvis. Its range was transversely across the hypogastrium, and from above downwards.—*South. Med. and Surg. Journ.*

